# Challenging the dry core paradigm: hydrated reactivity emerges within micellar cores

**DOI:** 10.1039/d6sc03380f

**Published:** 2026-05-26

**Authors:** Riliga Wu, Tongyue Wu, Weijiang Guan, Chao Lu

**Affiliations:** a State Key Laboratory of Chemical Resource Engineering, Beijing University of Chemical Technology Beijing 100029 China wjguan@mail.buct.edu.cn luchao@mail.buct.edu.cn; b Pingyuan Laboratory, College of Chemistry, Zhengzhou University Zhengzhou 450001 China

## Abstract

At the heart of every micelle lies a densely packed core that provides nanoscale confinement. However, the micellar core remains largely unexplored owing to its hydrophobic and inaccessible nature. Here, we overcome this long-standing obstacle by incorporating environment-responsive clusteroluminescent chromophores into micellar assemblies. These chromophores not only report the local core environment, but also activate the core *via* packing perturbation, yielding a environment-responsive, hydrated, and chemically accessible nanospace. Emission-based mapping reveals a marked environmental change upon micellization, indicating that the micellar core is moderately polar. Solubilization experiments monitored by Förster resonance energy transfer and solvent-addition studies analyzed by polarity mapping indicated that micellar interface expansion and core packing perturbation each facilitate water penetration into the micellar core. The hydroxide-mediated reaction further confirms the chemical accessibility of the micellar core, with a rate constant comparable to values reported for reactions at micellar interfaces. These findings suggest a feasible strategy for developing related dynamic, reactive, and self-reporting micellar nanospaces.

## Abstract 2

Embedding environment-responsive clusteroluminescent chromophores into micelles unlocks the long-assumed inert micellar core, revealing a polarity-sensitive, hydrated, and chemically reactive nanospace.

## Graphical abstract

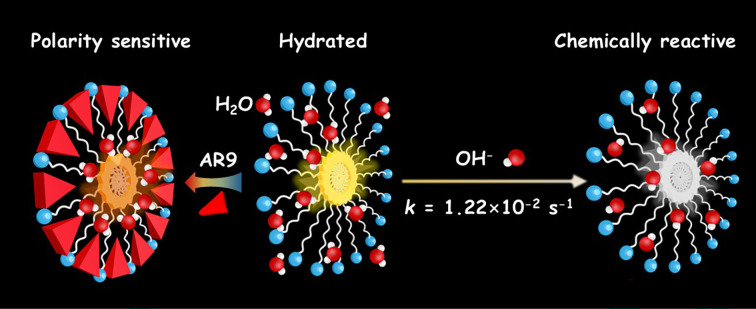

## Introduction

Micelles are fundamental supramolecular assemblies that underpin a wide range of chemical and biological processes.^1^ They provide nanoscopic reaction spaces, solubilization media, and model systems for understanding molecular organization in aqueous environments.^1–3^ Traditionally, micelles are viewed as a hydrated and reactive shell enclosing a dry and inert core.^4^ This dichotomy has shaped decades of micellar catalysis and encapsulation studies, where reactions and polarity changes are assumed to occur exclusively at or near the interface rather than within the core.^5^ Recently, emerging spectroscopic and scattering studies have begun to challenge this paradigm, revealing subtle yet reproducible signatures of water confinement within nominally hydrophobic interiors.^6–8^ These observations imply that micellar cores may contain dynamically bound water molecules that modulate local polarity and reactivity.^7–10^ If this is realizable, such hydration would fundamentally redefine the boundary between nanoconfinement and nanoscale chemistry, enabling inert micellar cores to serve as hydrated reactive domains. However, two key questions remain unresolved: how a hydrophobic core can accommodate water and manifest chemical reactivity, and how such hidden hydration and reactivity can be directly quantified.

Over the past decades, considerable efforts have been devoted to elucidating the internal microenvironment of micelles and related self-assembled systems.^11,12^ A wide range of analytical techniques, such as polarity-sensitive fluorescence probes, vibrational and nuclear magnetic resonance (NMR) spectroscopy, and small-angle X-ray scattering (SAXS), have revealed indirect indications of water penetration, local polarity fluctuations, and structural heterogeneity within hydrophobic interiors.^13–17^ However, these findings remain largely inferential: most fluorescent probes preferentially localize at the micellar interface rather than within the true core, while ensemble-averaged scattering or spectroscopic data obscure transient hydration and local reactivity.^18,19^ As a result, the precise extent and chemical relevance of confined water inside micellar cores are still poorly defined. Parallel to these probing efforts, modulation of micellar interiors has been explored using external triggers such as temperature, solvent polarity, light, redox potential, or surfactant composition to alter core packing and hydration.^20–26^ However, such modulation is typically inferred from macroscopic properties rather than from direct molecular-scale evidence.^27^ Consequently, the molecular basis of these modulation effects remains poorly defined, as current techniques largely cannot directly correlate structural changes with local hydration or reactivity inside the core.^28^ To bridge these gaps, a unified molecular strategy is needed to simultaneously report and regulate the hydration and reactivity of micellar cores *in situ*. Achieving such coupled sensing–modulation capability will transform micellar cores from passive nanocontainers into programmable nanospaces, offering a new platform to link nanoconfinement with emergent chemical function.

**Scheme 1:**
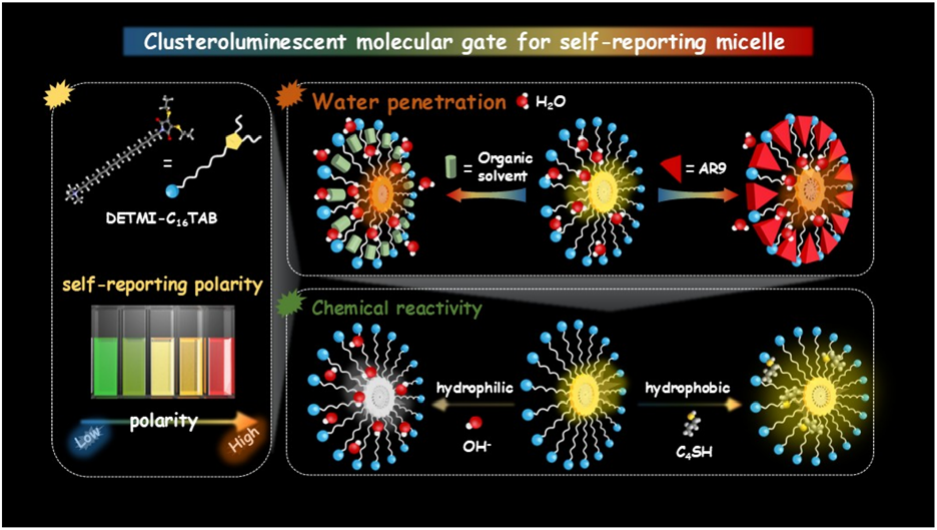
Schematic illustration of clusteroluminescent molecular gating that transforms dry inert micellar cores into hydrated reactive nanospaces.

To address these challenges, we introduce a self-reporting supramolecular gating strategy that integrates structural regulation and environmental sensing within a single molecular framework. Clusteroluminescence is a unique photophysical phenomenon arising from nonconjugated molecular clusters, which has been observed to show notable sensitivity to local polarity and confinement.^29,30^ By embedding clusteroluminescence units into micellar assemblies, the emission response of the clusters could serve as a self-reporting probe of the microenvironment, while their aggregation state serves as a molecular gate that governs the accessibility and hydration of the core. As a proof of concept, we covalently integrated a maleimide-derived clusteroluminescence chromophore (DETMI) at the end of a cetyltrimethylammonium (C_16_TAB) surfactant chain. The resulting DETMI-C_16_TAB forms micelles with environment-sensitive emission and intrinsic chemical reactivity, enabling multimodal monitoring of the micellar microenvironment. Nanosecond-resolved spectroscopic analyses revealed that the micellar core constitutes a moderately polar and dynamically relaxing domain. Spectroscopic investigations further indicated that solubilization expands the micellar interface to facilitate water penetration, whereas water-miscible organic solvents perturb micellar packing, inducing transient swelling followed by collapse at higher solvent fractions. To validate the inferred chemical accessibility of the micellar interior, penetration experiments with hydroxide ions and butanethiol confirmed that both hydrophilic and hydrophobic small molecules can diffuse into the micellar core (Scheme 1). This work presents a proof-of-concept design strategy for constructing self-reporting micellar systems, offering new opportunities for real-time tracking, adaptive regulation, and functional design of soft matter across nanoscience, materials chemistry, and biomedicine.

## Results and discussion

### Synthesis and characterization of DETMI-C_16_TAB

**Fig. 1:**
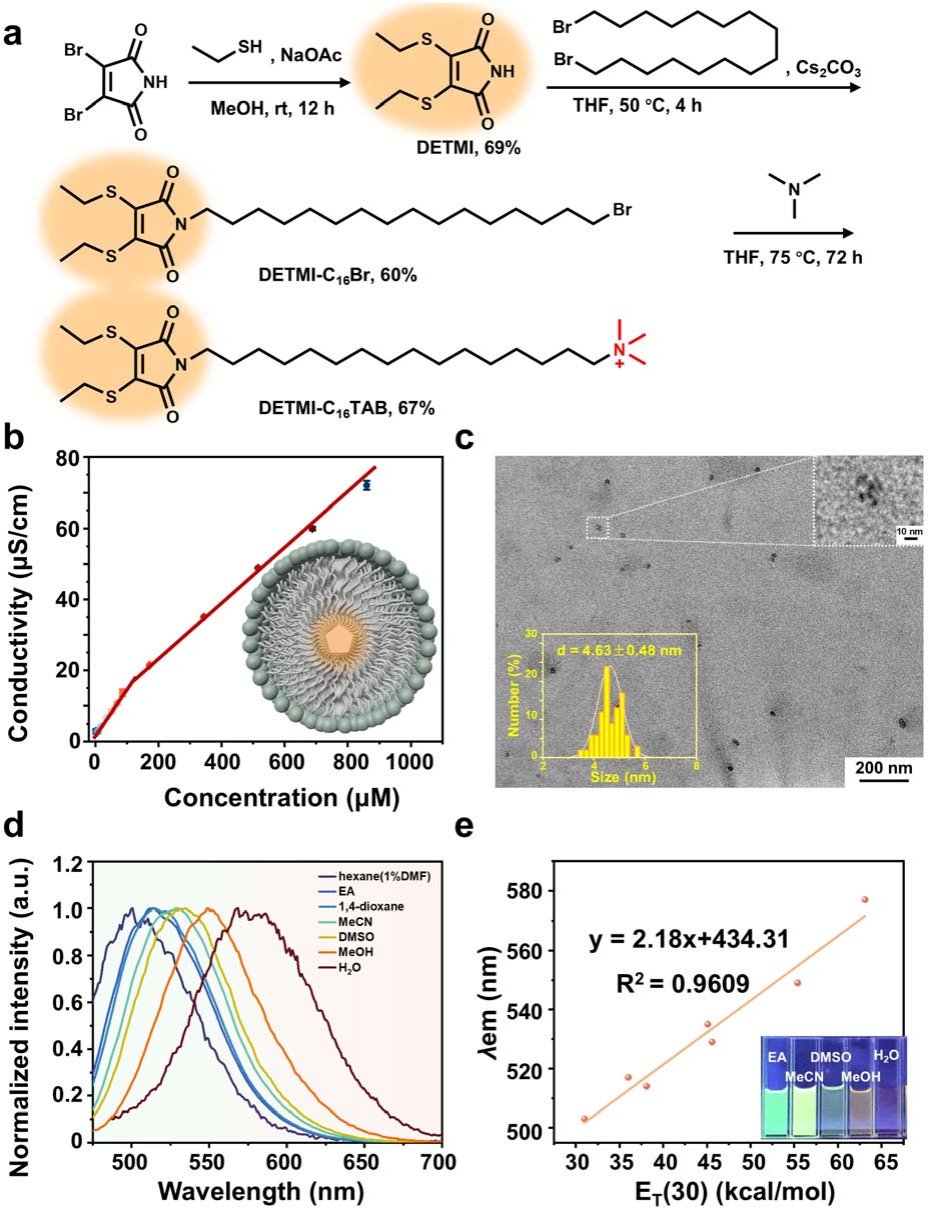
(a) Synthesis route of DETMI-C_16_TAB. (b) Plot of conductivity *versus* the concentration of DETMI-C_16_TAB (the inset shows a scheme of DETMI-C_16_TAB micelle with clusteroluminescence core). (c) HRTEM image of DETMI-C_16_TAB micelles (the inset shows the size distribution measured from 100 randomly selected particles). (d) Normalized fluorescence spectra of DETMI-C_16_TAB in different solvents. (e) Plot of maximum emission wavelength (*λ*_em_) of DETMI-C_16_TAB*versus* the solvent polarity parameter, *E*_T_(30), in different solvents.

The synthesis and structural characterization of DETMI-C_16_TAB (Fig. 1a) were first carried out to confirm its molecular identity. As illustrated in Fig. 1a, the synthesis began with the preparation of the clusteroluminescence DETMI core through the reaction of dibromomaleimide with ethanethiol.^31^ This was followed by alkylation with 1,16-dibromohexadecane to yield the intermediate DETMI-C_16_Br. The chemical structures of DETMI (Fig. S1) and DETMI-C_16_Br (Fig. S2) were confirmed by proton nuclear magnetic resonance (^1^H NMR) spectroscopy. Subsequently, DETMI-C_16_Br was quaternized with an excess of trimethylamine to afford the final product, DETMI-C_16_TAB, which was verified by both ^1^H NMR (Fig. S3) and ^13^C NMR spectroscopy (Fig. S4). The molecular identity was further corroborated by positive-ion mass spectrometry, which exhibited an *m*/*z* ratio of 499.3385. This is in agreement with the calculated theoretical value of 499.3386 (Fig. S5). These results demonstrate the successful preparation of DETMI-C_16_TAB as a structurally well-defined surfactant for subsequent studies of its self-assembly and photophysical behaviors.

The self-assembly behavior of DETMI-C_16_TAB in aqueous solution was subsequently investigated through determination of its critical micelle concentration (CMC) and micellar morphology. Using the conductivity method, an inflection point on the conductivity–concentration curve indicated a CMC value of about (134 ± 14) µM (Fig. 1b). Linear fitting of the conductivity data below and above the CMC yielded *k*_2_/*k*_1_ (post-/pre-CMC) values of 0.61 for DETMI-C_16_TAB and 0.25 for C_16_TAB, indicating that DETMI-C_16_TAB micelles undergo weaker counterion binding than C_16_TAB micelles.^32^ High-resolution transmission electron microscopy (HRTEM) image revealed the presence of spherical micelles with an average diameter of 4.63 ± 0.48 nm (Fig. 1c). Consistently, dynamic light scattering (DLS) analysis further confirmed the formation of nanosized aggregates with an average hydrodynamic diameter of 5.00 ± 0.06 nm (Fig. S6). These results demonstrate that DETMI-C_16_TAB forms spherical micelles in water. Due to the presence of the solvation layer, the hydrodynamic diameter is larger than the diameter determined by HRTEM.

The photophysical properties of DETMI-C_16_TAB were further examined to evaluate its polarity-dependent fluorescence behavior. As the fluorescence of DETMI clusters has been reported to be sensitive to the polarity of their surrounding environment,^33^ the amphiphilic DETMI-C_16_TAB was expected to exhibit a similar response. Fluorescence emission spectra recorded in solvents of varying polarity revealed a progressive red shift of the emission maximum from 503 to 577 nm (Fig. 1d). DETMI-C_16_TAB shows a slight overall red shift in emission wavelength relative to DETMI.^31^ To quantitatively evaluate this solvent effect, the emission maxima were correlated with the empirical solvent polarity parameter *E*_T_(30).^34^ A strong positive correlation (*R*^2^ = 0.9609) was observed over the *E*_T_(30) range of 31–63.1 (Table S1 and Fig. 1e), providing a semi-quantitative description of the polarity-dependent fluorescence behavior. On the other hand, ultraviolet-visible (UV-vis) absorption spectra displayed negligible changes in either peak position or absorbance upon varying the solvent (Fig. S7). Thus, the observed polarity-dependent fluorescence primarily arises from changes in excited-state interactions, indicating the sensitivity of DETMI-C_16_TAB to local microenvironments in self-assembled systems. In addition, upon continuous irradiation at 410 nm using a xenon lamp, DETMI-C_16_TAB can retain 98% of its initial emission intensity after 1 h (Fig. S8). These results confirm that DETMI-C_16_TAB is a promising and photostable probe for monitoring micellar microenvironments and related dynamic processes.

### Fluorescence monitoring of micelle formation

**Fig. 2:**
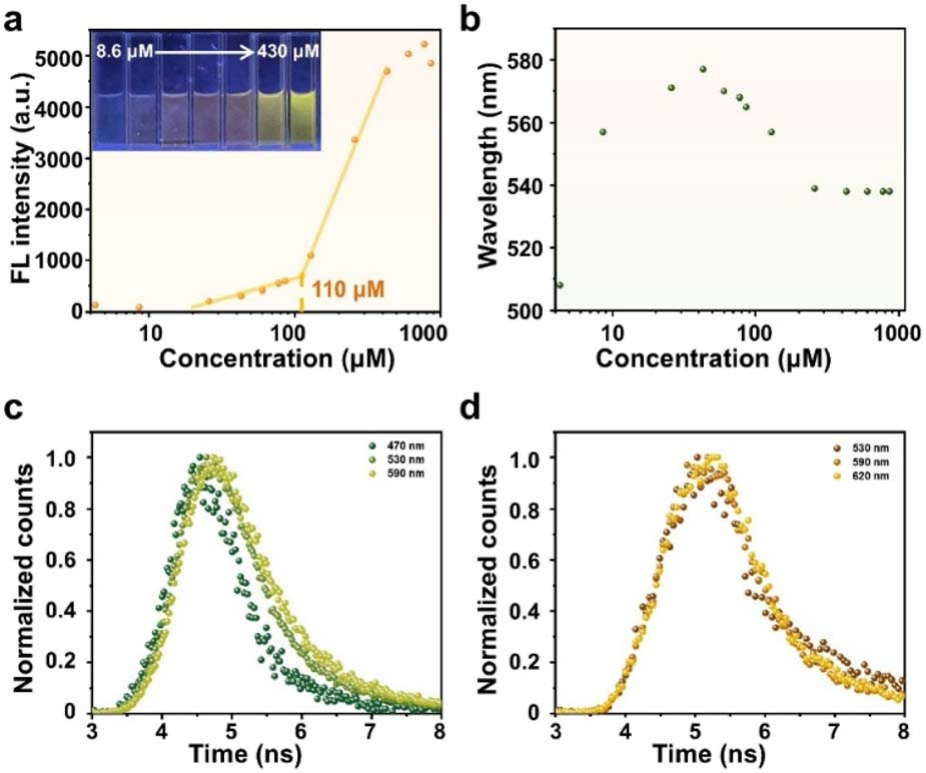
Plots of (a) fluorescence (FL) intensity and (b) fluorescence wavelength *versus* the concentration of DETMI-C_16_TAB in the aqueous phase. (c) Fluorescence decays of DETMI-C_16_TAB in (c) micellar and (d) non-micellar states.

The polarity-dependent fluorescence of DETMI-C_16_TAB enables direct monitoring of the micellization process as a function of surfactant concentration. As shown in Fig. S9, the fluorescence intensity increased progressively over the concentration range of 4.3–860 µM, with a distinct inflection point at about 110 µM (Fig. 2a), in good agreement with the CMC determined by conductivity measurements. This increase in fluorescence suggests that micelle formation promotes aggregation of the tail-associated DETMI moieties to induce clusteroluminescence. Concomitantly, the emission maximum of DETMI-C_16_TAB exhibited a red shift at concentrations below 43 µM, followed by a blue shift from 577 nm at 43 µM to 538 nm at 258 µM, after which the emission maximum stabilized at 538 nm with further increases in concentration (Fig. 2b). The initial red shift can be attributed to the formation of pre-micellar DETMI-C_16_TAB clusters in the aqueous phase prior to micelle formation.^35^ The subsequent blue shift is ascribed to micellization, where DETMI moieties experience a relatively less polar environment within the micellar interior compared to bulk water.

The established correlation between the emission maximum and the *E*_T_(30) value (Fig. 1e) enables a semi-quantitative assessment of the micellar polarity. As shown in Fig. 2b, the emission at 538 nm observed in the micellar state (above the CMC) corresponds to an *E*_T_(30) value of approximately 47.4 ± 0.3, which is comparable to that of DMSO (*E*_T_(30) = 45.1). This comparison suggests that the micellar interior exhibits a moderately polar environment rather than being strictly hydrophobic. The previously reported *E*_T_(30) values for probes located at different regions of C_16_TAB micelles are as follows: octanal, which is located closest to the aqueous phase, shows an *E*_T_(30) value of about 48.6; di-*n*-hexyl ketone, located closest to the micellar core, gives a value of about 36.0; and 1-naphthaldehyde, positioned between these two regions, shows a value of about 45.0.^6^ While these fluorescence results revealed the sensitivity of the excited state to micellar polarity, the ground-state absorption showed no detectable spectral shift upon micellization (Fig. S10). Moreover, the absorbance at 410 nm exhibited a good linear correlation (*R*^2^ = 0.9996) with concentration across the same range (Fig. S11), indicating that the micellar environment exerts negligible influence on the ground-state absorption properties. Fig. S12 shows the UV-vis spectra of DETMI in C_16_TAB solution at different concentrations. The absorbance shows a linear dependence on concentration, with an *R*^2^ value of 0.9995 (Fig. S13), indicating well-behaved absorption over the studied concentration range. This trend is consistent with that observed for DETMI-C_16_TAB. These findings indicate that the micellar interior possesses a moderately polar environment.

To further investigate the effect of hydrogen bonding on the emission wavelength, we performed a multiple linear regression using the Kamlet–Taft linear solvation energy relationship, *ν*_max_ = *ν*_0_ + *aα* + *bβ* + *s*π*, in order to evaluate the contributions of solvent hydrogen-bond donor acidity (*α*), hydrogen-bond acceptor basicity (*β*), and solvent polarity/polarizability (π*). In the full model, the *β* term was not statistically significant (*P* > 0.05) and was therefore excluded from the reduced fit. The final equation, *ν*_max_ = 19 923.0 − 1193.2*α* − 1044.8π* (cm^−1^), gave a good correlation (*R*^2^ = 0.9682; Tables S2 and S3). These results indicate that both *α* and π* contribute to the observed emission shift.^36^ Therefore, the observed behavior is regarded as an empirical measure of the local solvation environment of the micellar microenvironment, which may include contributions from both polarity/polarizability and hydrogen-bonding interactions.

To gain further insight into the micellar polarity, fluorescence decays of DETMI-C_16_TAB were recorded at different emission wavelengths under micellar and non-micellar conditions. As shown in Fig. 2c, in the micellar state above the CMC, the fluorescence decays of DETMI-C_16_TAB are emission-wavelength-dependent. The decay is faster at the blue edge of the fluorescence spectrum (470 nm), where it mainly arises from emission of unsolvated dipoles formed in the excited state without undergoing relaxation. In contrast, the decay is slower at the red edge of the spectrum (590 nm), indicating that the excited-state dipoles undergo solvent relaxation prior to fluorescence emission, resulting in a delayed decay. This behavior suggests that under micellar conditions, a fraction of DETMI-C_16_TAB molecules is located in hydrated regions of the micelles, where solvent relaxation can occur. In comparison, if the dynamics occurred solely in bulk water, they would be too fast to be resolved by the time-correlated single-photon counting (TCSPC) setup used in this work.^18,19^ As expected, in the non-micellar state below the CMC, the fluorescence decays of DETMI-C_16_TAB are independent of emission wavelength (Fig. 2d). Therefore, the wavelength-dependent fluorescence decays provide dynamic evidence for the hydrated micellar environment, consistent with the moderate polarity inferred from fluorescence-polarity correlation studies. The measured lifetimes are 0.8 ns in the micellar state and 1.3 ns in the non-micellar state. These results suggest that aggregation within the micellar core modifies the excited-state decay behavior, reflecting a change in the local microenvironment.

### Fluorescence probing of water penetration

While micelle formation inherently permits a certain degree of hydration, it remains unclear under what conditions water molecules are able to penetrate deeper into the micellar interior. Elucidating this aspect is crucial, as the extent of water penetration governs not only the structural flexibility of micelles but also their capacity to mediate solubilization processes. To address this, solubilization experiments were carried out and studied by Förster resonance energy transfer (FRET) analysis. DETMI moieties anchored to the hydrophobic tails of C_16_TAB were chosen as the FRET donor. Acid Red 9 (AR9), a non-fluorescent dye with an absorption spectrum that overlaps well with the emission spectrum of DETMI, was selected as the FRET acceptor. In this design, changes in the emission spectra originate exclusively from the donor, thus avoiding potential interference from acceptor emission.

**Fig. 3:**
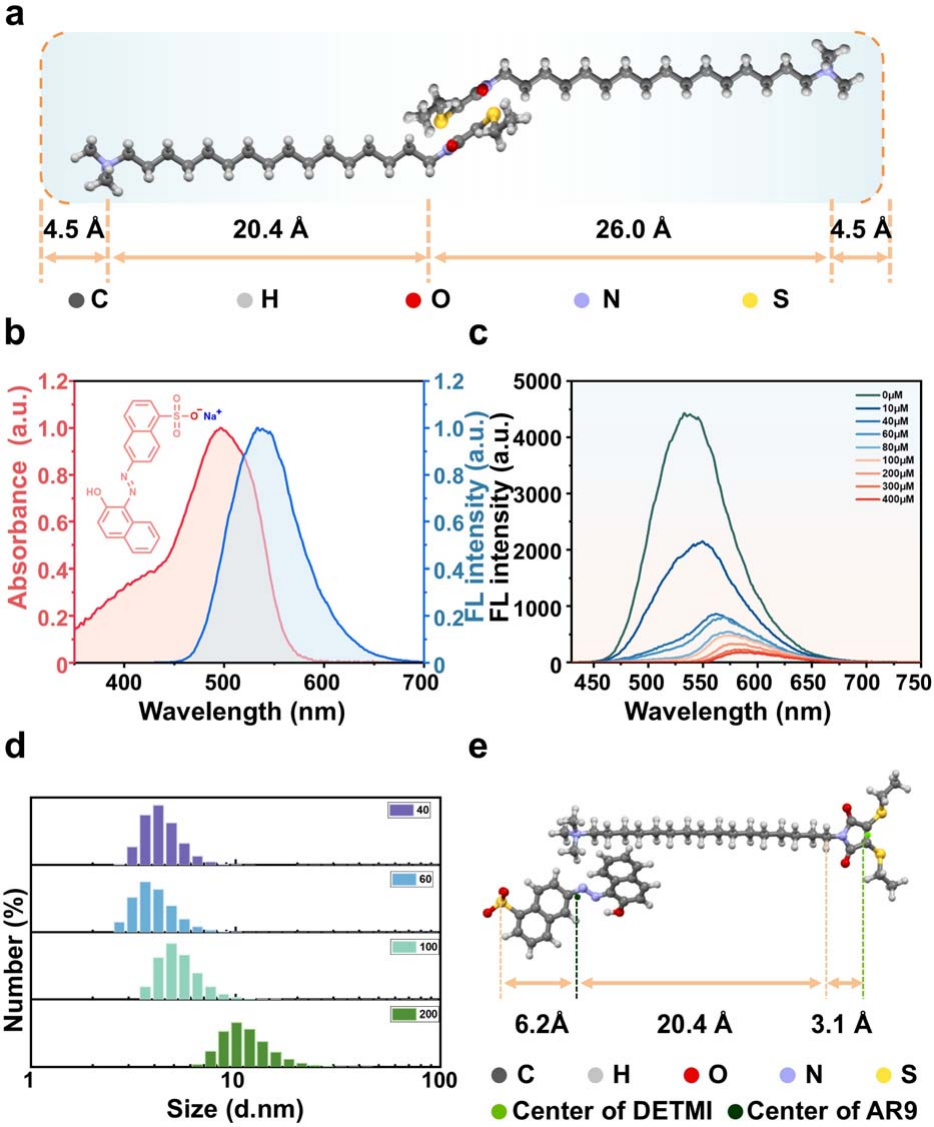
(a) Energy-minimized molecular structure of DETMI-C_16_TAB. (b) Normalized emission spectra of DETMI-C_16_TAB micelle (blue region) and normalized absorption spectra of AR9 (red region). (c) Fluorescence spectra of DETMI-C_16_TAB micelle in the absence and presence of AR9 with different concentrations. (d) DLS data of DETMI-C_16_TAB micelle in the presence of AR9 with different concentrations. (e) Calculation of the distance between the AR9 and DETMI luminescent centers.

To estimate the DETMI position within the micelle, the geometry of DETMI-C_16_TAB was optimized using density functional theory (DFT) at the B3LYP/6-311G(d,p) level using the Beijing Density Functional (BDF) program.^37–41^ Interatomic distances of DETMI-C_16_TAB were estimated from the DFT-optimized geometry using Mercury software. As shown in Fig. 3a, the distance between the ammonium nitrogen atom and the terminal carbon atom is ∼26.0 Å. The solvation layer around cationic headgroups typically contributes an additional thickness of ∼4.5 Å.^42^ Lattice packing analysis suggested that neighboring DETMI moieties could adopt a face-to-face stacked arrangement with an overlapped length of ∼5.6 Å. Following a simple geometrical estimate commonly used for ionic micelles, the micelle diameter can be approximated by the sum of two effective surfactant lengths (radially oriented) plus a hydration/shell thickness term that accounts for the headgroup region, counterion binding and bound water:*D*_est_ ≈ 2*L*_eff_ − *Δ*_overlap_ + 2*δ*_hyd_where *L*_eff_ accounts for the molecular length along the radial direction (alkyl segment plus the contribution of the DETMI unit and headgroup region), *Δ*_overlap_ ≈ 5.6 Å reflects the fully overlapped DETMI stacking, and *δ*_hyd_ ≈ 4.5 Å is the hydration thickness. Using the dimensions extracted from the DFT-optimized structure, this yields *D*_est_ ≈ 55.4 Å. This geometrical estimate is comparable in magnitude to the hydrodynamic diameter determined by DLS (∼5.00 nm), supporting that DETMI moieties can be accommodated within the micellar interior.

The FRET donor–acceptor distance was calculated using eqn (1) and (2):

1 *E* = *R*_0_^6^/(*R*_0_^6^ + *r*^6^)

2 *R*_0_ = 0.211(*κ*^2^*n*^−4^*Q*_D_*J*(*λ*))^−6^

where *R*_0_ is the Förster distance, *r* is the distance between the centers of the donor and acceptor, *κ*^2^ is the orientation factor, *n* is the refractive index of the medium, *Q*_D_ is the fluorescence quantum yield of the donor, and *J*(*λ*) is the spectral overlap integral derived from Fig. 3b.^43^

The FRET efficiency (*E*) was obtained according to eqn (3) (Fig. S14):

3 *E* = 1 − (*I*_DA_/*I*_D_)

where *I*_DA_ and *I*_D_ represent the donor emission intensities in the presence and absence of acceptor, respectively. As shown in Fig. 3c, incremental addition of AR9 (0–400 µM) led to a gradual decrease in the fluorescence intensity of DETMI-C_16_TAB, accompanied by a red shift of the emission peak to 577 nm. The decrease in intensity confirmed efficient FRET. The accompanying red shift suggested that the microenvironment surrounding DETMI within the micellar core became increasingly polar, resembling that of water. DLS measurements (Fig. 3d) further revealed that the micellar size remained unchanged at ∼100 µM AR9, but increased to ∼20 nm at 200 µM AR9, consistent with micellar expansion. To ensure that FRET occurred within intact micelles, the efficiency value obtained at 100 µM AR9 (98.03%) was selected for distance calculations, yielding a donor–acceptor separation of (23.75 ± 0.03) Å (Fig. 3e). These results suggest that solubilization can promote micellar interface expansion and increase the accessibility of water molecules into the micellar core.

To further illustrate the comparison of accessibility between DETMI-C_16_TAB micelle and traditional surfactant micelles, Nile red was used as a polarity-responsive probe to compare DETMI-C_16_TAB and C_16_TAB micelles (Fig. S15). The results showed that the emission maximum of Nile red occurred at a longer wavelength in C_16_TAB micelles than in DETMI-C_16_TAB micelles, suggesting a difference in the local microenvironment in the two micellar systems.

### Fluorescence probing of organic solvent penetration

**Fig. 4:**
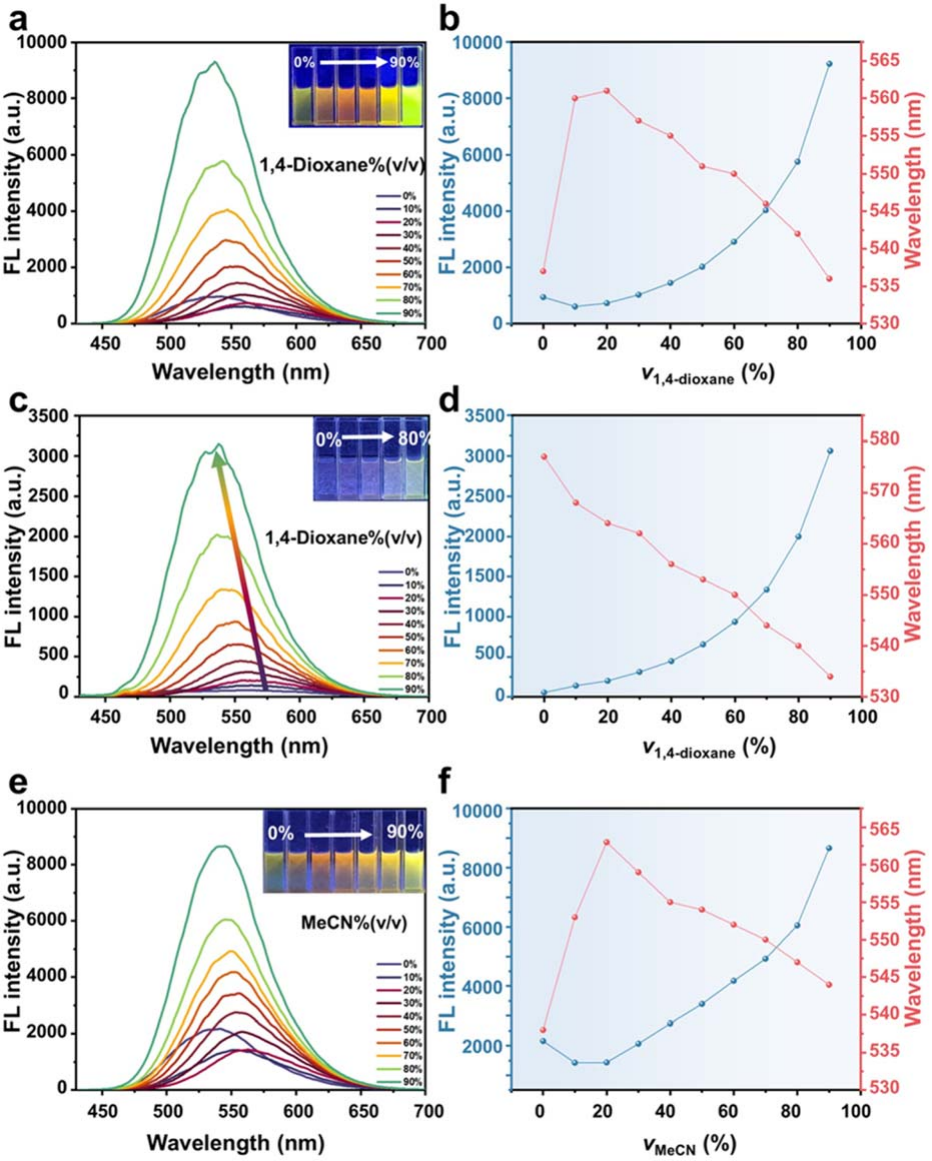
Fluorescence emission spectra and plot of FL intensity and wavelength *versus* the volume of 1,4-dioxane of (a and b) DETMI-C_16_TAB micelles and (c and d) disassociate DETMI-C_16_TAB in 1,4-dioxane/water mixtures with different 1,4-dioxane fractions. Fluorescence emission spectra and plot of FL intensity and wavelength *versus* the volume of solvents of DETMI-C_16_TAB micelles in (e and f) acetonitrile/water mixtures with different acetonitrile fractions.

Building upon the understanding of water penetration facilitated by solubilization, the study was extended by introducing water-miscible organic solvents. This approach enabled a systematic evaluation of how variations in micellar packing and polarity influence the accessibility of water molecules to the core regions. Two representative solvents, 1,4-dioxane and acetonitrile, were chosen for their comparable miscibility with water but contrasting polarity.^44^ As shown in Fig. 4a, upon gradual addition of 1,4-dioxane (a weakly polar solvent), the emission of DETMI-C_16_TAB micelles first red-shifted from 538 to 561 nm with decreased intensity (0–20%, v/v), suggesting that partial solvent penetration promoted micelle swelling and exposed the DETMI chromophores to a more polar, water-rich environment. At higher 1,4-dioxane fractions (20–90%, v/v), however, the emission blue-shifted back to 536 nm with a concomitant intensity increase, suggesting the micelle collapse and direct exposure of DETMI chromophores to the less polar dioxane/water mixture (Fig. 4b). This interpretation is supported by DLS analysis, which revealed micelle swelling up to ∼20 nm at 20% v/v (Fig. S16). In contrast, when the same solvent gradient was applied to a dilute solution of DETMI-C_16_TAB (43 µM, below the CMC), only a monotonic blue shift (577–537 nm) with increasing intensity was observed (Fig. 4c and d), indicating a continuous decrease in polarity in the absence of micellar protection.

Acetonitrile (a moderately polar solvent) induced a similar biphasic spectral response (Fig. 4e). A red shift from 538 to 563 nm occurred at low acetonitrile fractions (0–20%, v/v), attributable to micelle swelling and enhanced water accessibility of the DETMI chromophores (Fig. 4f). At higher acetonitrile fractions (20–90%, v/v), the emission gradually blue-shifted to 544 nm with an increased intensity, in line with DLS evidence of micelle disruption (Fig. S17). Our results semi-quantitatively capture solvent-induced changes in micellar packing. At low solvent fractions, increased water accessibility and transient swelling are observed, whereas micellar collapse occurs at high solvent fractions. These findings are consistent with previously reported micellar behavior and validate the reliability and sensitivity of our self-reporting platform for probing small-molecule diffusion in this microenvironment.

### Chemical reactivity of micellar core

**Fig. 5:**
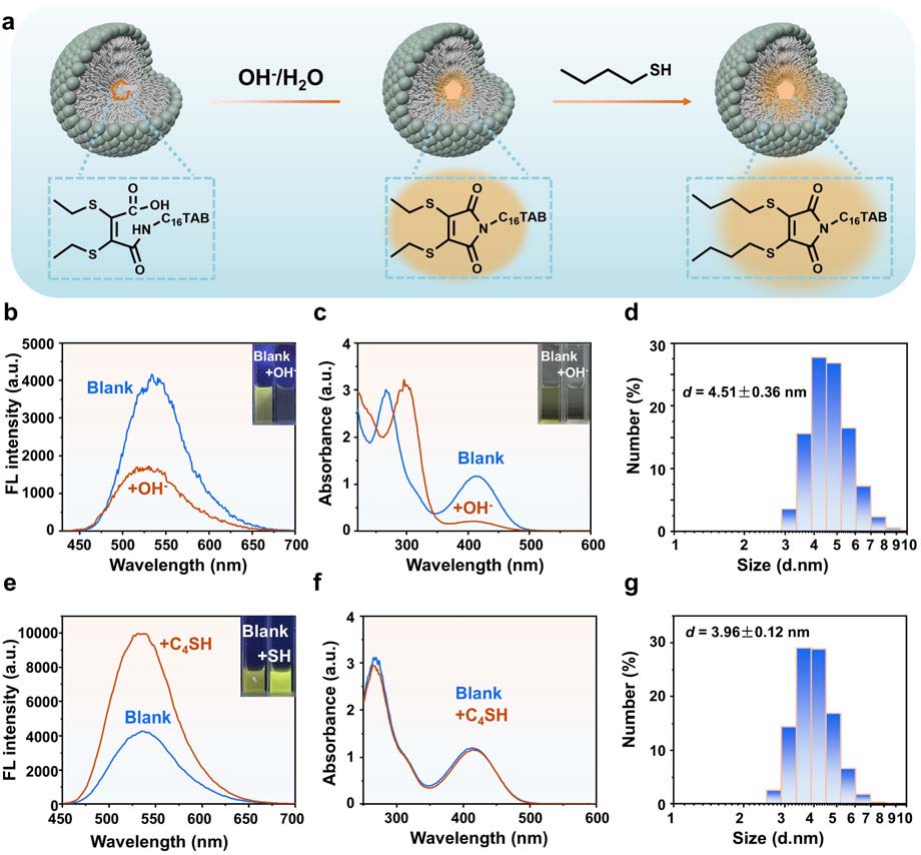
(a) Scheme of the reaction between DETMI and hydrophilic hydroxide ions (OH^−^) as well as hydrophobic *n*-butyl mercaptan (C_4_SH). (b) Fluorescence emission spectra, (c) UV-vis spectra and (d) DLS data of DETMI-C_16_TAB micelles at addition of OH^−^. (e) Fluorescence emission spectra, (f) UV-vis spectra and (g) DLS data of DETMI-C_16_TAB micelles with addition of C_4_SH.

The broader functional significance of micelles in chemical and biological processes depends critically on their ability to host and exchange small molecules within the interior. By probing the accessibility of the micellar core to both hydrophilic and hydrophobic reactive species, it is possible to provide essential insight into the chemical reactivity and transport phenomena mediated by micelles. Herein, the hydrophilic hydroxide ions (OH^−^) and hydrophobic *n*-butanethiol (C_4_SH) were employed as reactive probes (Fig. 5a).^45,46^ As 500 µM NaOH was introduced to the micellar system, the solution color faded from yellow to colorless, accompanied by the disappearance of characteristic UV-vis absorption peaks at 270 nm and 410 nm (Fig. 5b), indicating the ring-opening hydrolysis of maleimide groups of DETMI. Consistently, fluorescence intensity decreased with the addition of OH^−^ (Fig. 5c), and MS analysis (*m*/*z* = 517.3488) confirmed the hydrolyzed product (Fig. S18). DLS measurements revealed that the micelle size remained approximately 4 nm in the presence of 500 µM NaOH (Fig. 5d), indicating that OH^−^ ions were able to penetrate the micelle and react with the DETMI core without compromising micellar integrity. Based on this observation, the pseudo-first-order rate constant (*k*) for the reaction was further determined. As shown in Fig. S19, the absorbance (*A*) at 410 nm gradually decreased with increasing reaction time, reflecting the progression of the reaction and the consumption of the DETMI moiety. After approximately 300 s, the absorption reached a plateau and no further decrease was observed. Linear fitting of ln(*A*_*t*_ − *A*_∞_) *versus* time yielded a rate constant of (1.220 ± 0.007) × 10^−2^ s^−1^ (Fig. S20). This value is comparable to values reported (Table S4) for hydroxide-mediated reactions at micellar interfaces,^47^ indicating efficient hydroxide access to the micellar core.^48–51^

On the other hand, the addition of 1 mM C_4_SH showed a 2.3-fold enhancement in fluorescence intensity (Fig. 5e), while the UV-vis spectrum remained unchanged (Fig. 5f). This suggests a thiol-exchange reaction occurred at the micellar core without altering the chromophore structure of DETMI. MS data (*m*/*z* = 555.4014) confirmed the formation of the exchange product (Fig. S21), and DLS analysis showed unchanged micelle size (∼4 nm) after reaction (Fig. 5g), implying that C_4_SH successfully entered the micelle core and reacted with DETMI without compromising micelle stability. For comparison, after reaction with C_4_SH, the fluorescence intensity increases (Fig. S22) whereas the UV-vis absorption spectrum remains largely unchanged (Fig. S23). This trend is consistent with the spectral behavior observed for DETMI-C_16_TAB micelles in the presence of C_4_SH, and thus strengthens our interpretation that the thiol-exchange process primarily influences the emissive properties without substantially changing the ground-state chromophore framework. These findings demonstrate that both hydrophilic and hydrophobic small molecules can diffuse into the micelle core, revealing its structural permeability and offering insights for designing responsive micellar systems.

## Conclusions

In summary, we established a self-reporting micellar platform that both optically reveals and functionally activates the micellar core, transforming it from a conventionally presumed inert hydrophobic pocket into a hydrated and reactive nanospace. The correlation between emission maxima and environment enabled a semi-quantitative assessment of core environment, showing that the micellar core is moderately polar. Time-resolved spectroscopy further demonstrated that the core is dynamically relaxing and accessible to water molecules. Water accessibility within the micelle arises from two mechanisms: micellar interface expansion, which promotes water access at the interfacial region, and perturbation of hydrophobic core packing, which enables deeper water penetration into the core. Reaction-based penetration studies confirmed that both hydrophilic and hydrophobic small molecules can access and react within the micellar core, confirming its chemical accessibility and reactivity. These findings redefine the DETMI-C_16_TAB micellar core as a structurally dynamic and chemically active nanospace. More importantly, the integration of environment-responsive chromophores establishes a general design principle for self-reporting supramolecular systems, providing a versatile platform for real-time visualization and functional control of soft materials.

## Author contributions

Weijiang Guan conceived the experiments. Riliga Wu and Tongyue Wu carried out the experiments. Riliga Wu, Weijiang Guan and Chao Lu contributed to the data analysis and writing of this manuscript.

## Conflicts of interest

There are no conflicts to declare.

## Supplementary Material

SC-OLF-D6SC03380F-s001

SC-OLF-D6SC03380F-s002

## Data Availability

The data supporting this article have been included as part of the supplementary information (SI). Supplementary information is available. See DOI: https://doi.org/10.1039/d6sc03380f.
