# Supplementary material for: Challenging the dry core paradigm: hydrated reactivity emerges within micellar cores
Source: Chem Sci. 2026 May 26;17(24):11874–81. doi: 10.1039/d6sc03380f (PMC13207891; doi:10.1039/d6sc03380f)
Supplement: SC-017-D6SC03380F-s002 [file SC-017-D6SC03380F-s002.pdf]

Supplementary Information (SI) for Chemical Science.
This journal is © The Royal Society of Chemistry 2026

Supporting Information for

Challenging the Dry Core Paradigm: Hydrated Reactivity Emerges within Micellar Cores

Riliga Wu,[†] Tongyue Wu,[†] Weijiang Guan*,[†] and Chao Lu*,[†,‡]

[†]*State Key Laboratory of Chemical Resource Engineering, Beijing University of Chemical Technology, Beijing 100029, China.*

[‡]*Pingyuan Laboratory, College of Chemistry, Zhengzhou University, Zhengzhou 450001, China.*

*E-mail: wjguan@mail.buct.edu.cn

*E-mail: luchao@mail.buct.edu.cn

Contents

Figure S1. ^{1}H NMR spectrum of DETMI in $CDCl_3$.

Figure S2. ^{1}H NMR spectrum of DETMI-C_{16}Br in $CDCl_3$.

Figure S3. ^{1}H NMR spectrum of DETMI-C_{16}TAB in CD_3OD.

Figure S4. ^{13}C NMR spectrum of DETMI-C_{16}TAB in CD_3OD.

Figure S5. Magnification of the positive-ion mass spectrum of DETMI-C_{16}TAB.

Figure S6. DLS of DETMI-C_{16}TAB micelle.

Figure S7. Absorption spectra of DETMI-C_{16}TAB with 43 μM in different solvents.

Figure S8. Photostability of the DETMI-C_{16}TAB aqueous solution (43 μM) under continuous irradiation at 410 nm using a xenon lamp.

Figure S9. Fluorescence emission spectra of DETMI-C_{16}TAB with different concentrations in water.

Figure S10. Absorption spectra of DETMI-C_{16}TAB with different concentrations in water.

Figure S11. Plot of absorbance versus the concentrations of DETMI-C_{16}TAB.

Figure S12. Absorption spectra of DETMI with different concentrations in C_{16}TAB micelle.

Figure S13. Plot of absorbance versus the concentrations of DETMI in C_{16}TAB micelle.

Figure S14. FRET efficiency of DETMI-C_{16}TAB micelle in the presence of AR9 with different concentrations.

Figure S15. Normalized fluorescence emission spectra of Nile Red in DETMI-C_{16}TAB micelles, C_{16}TAB micelles and water.

Figure S16. DLS of DETMI-C_{16}TAB micelle in 1,4-dioxane/water mixtures with 20% of 1,4-dioxane fractions.

Figure S17. DLS of DETMI-C_{16}TAB micelle in acetonitrile /water mixtures with 20% of acetonitrile fractions (*f*w).

Figure S18. Positive-ion mass spectrum of the reaction product of DETMI-C_{16}TAB and OH^-.

Figure S19. Kinetic traces for hydrolysis of DETMI-C_{16}TAB.

Figure S20. Plot of ln(*A*) as a function of time for the degradation of DETMI-C_{16}TAB. The pseudo-first order rate constant *k* was determined from the slope of the linear regression line.

Figure S21. Positive-ion mass spectrum of the reaction product of DETMI-C_{16}TAB and

C_4SH.

Figure S22. Fluorescence emission spectra of DETMI with addition of C_4SH.

Figure S23. Absorption spectra of DETMI with addition of C_4SH.

Table S1. Maximum emission wavelength of DETMI-C_{16}TAB in different solvents.

Table S2. Solvent parameters (π^* and α) and maximum emission wavelength of DETMI-C_{16}TAB in different solvents.

Table S3. Multiple linear regression results for the Kamlet–Taft solvatochromic analysis of DETMI-C_{16}TAB

Table S4. The pseudo-first-order rate constant (k) for C_{16}TAB micelle-catalyzed hydrolysis reactions in the relevant literature.

Chemicals and Materials. All chemicals utilized in the experiments were of analytical reagent grade and were employed as received, without undergoing any further purification steps. 2,3-Dibromomaleinimide was procured from Bide Pharmatech Ltd. (Shanghai, China), while sodium acetate trihydrate, NaOH and NaCl was obtained from Xilong Chemical Industry Incorporated Co., Ltd. (Shenzhen, China). Ethanethiol was sourced from Acros Organics (Shanghai, China). 1,16-Dibromohexadecane was purchased from Shanghai Macklin Biochemical Co., Ltd. (Shanghai, China). Trimethylamine (2 M in THF) was purchased from Huawei Ruike Chemical Technology Co. (Beijing, China). Additionally, ethyl acetate, n-hexane, dichloromethane and methanol for column chromatography were acquired from Beijing Chemical Reagent Company (Beijing, China). Cesium carbonate was purchased from J&K Scientific Ltd. (Beijing, China). Tetrahydrofuran (THF), butanethiol (C_4SH), acetonitrile, dimethyl sulfoxide (DMSO), ethyl acetate, n-hexane, 1,4-dioxane, dichloromethane, N, N-dimethylformamide (DMF), ethanol and methanol for spectral analysis were obtained from energy chemical Pharmaceutical & Chemical Co. (Shanghai, China). Acid red 9 (AR9) was sourced from Tokyo Chemical Industry Co., Ltd. (Tokyo, Japan). Deionized water used in experiments was purified with a Milli-Q Advantage A10 (Merck Millipore, Germany).

Apparatus and Characterization. Proton and carbon-13 nuclear magnetic resonance (^1H NMR ^{13}C NMR) data were acquired employing a Bruker AV 600 NMR spectrometer (Bruker, Germany). Mass spectra (MS) were performed on a Quattro micro-triple quadrupole mass spectrometer (Waters, USA). Electrical conductivity measurements were carried out using a FE-30 conductivity meter (Mettler-Toledo, Switzerland). Dynamic light scattering (DLS) experiments were conducted using a Zetasizer Nano ZS (Malvern, UK). Fluorescence spectra were obtained utilizing an F-7000 fluorescence spectrophotometer (Hitachi, Japan) with a slit width of 5.0 nm and a scanning rate of 2400 nm/min. High-resolution transmission electron

micrographs were recorded on a JEOL JEM2010 200kV transmission electron microscope (JEOL, Japan). Absorption spectra were recorded on a U-3900H spectrometer (Hitachi, Japan). Time-resolved emission spectra were obtained by a FLS 980 fluorescence spectrophotometer (Edinburgh, UK).

Synthesis of DETMI. A mixture comprising 1.27 g of 2,3-dibromomaleinimide (5 mmol), 1.50 g of sodium acetate trihydrate (11 mmol), and 30 mL of methanol was combined in a 100 mL two-necked flask. Subsequently, 815 μL of ethanethiol (0.68 g, 11 mmol) was introduced into the aforementioned mixture, which was then stirred for 12 hours at room temperature. The resulting product underwent purification via column chromatography, utilizing hexane/ethyl acetate (6:1 *v/v*) as the eluent. The purified product was obtained as a yellow solid, yielding 69%. ^{1}H NMR (600 MHz, $CDCl_3$, δ): 7.41 (s, 1H), 3.31-3.33 (q, 4H), 1.33-1.36 (t, 6H).

Synthesis of DETMI-C_{16}Br. Cs_2CO_3 (2.9324 g, 9 mmol) and 1,16-dibromohexadecane (3.4587 g, 9 mmol) were added to the solution of DETMI (0.651 g, 3 mmol) in THF (60 mL), and the mixture was heated to 50 °C with stirring for 4 h. After cooling to room temperature, the mixture was filtered for removal of solid Cs_2CO_3. The residue was purified on a silica-gel column using n-hexane/dichloromethane (2:1 v/v) as eluent. DETMI-C_{16}Br was obtained as a yellow solid in 60% yield. ^{1}H NMR (600 MHz, $CDCl_3$, δ): 3.47-3.50 (t, 2H), 3.40-3.42 (t, 2H), 3.29-3.33 (q, 4H), 1.84-1.86 (m, 2H), 1.55-1.57 (m, 2H), 1.25-1.42 (m, 30H).

Synthesis of DETMI-C_{16}TAB. DETMI-C_{16}Br (0.1817g, 0.35 mmol) was added to the solution of trimethylamine in THF (2 M, 3.5 mL). The mixture was stirred and heated at 75 °C for 72 h. After the evaporation of THF and extra trimethylamine, the residue was purified by column chromatography using dichloromethane/methanol (25:1 v/v) as eluent. DETMI-C_{16}TAB was obtained as a yellow solid in 67% yield. ^{1}H NMR (600 MHz, CD_3OD, δ): 3.45-

3.54 (m, 2H), 3.30-3.37 (m, 6H), 3.15 (s, 9H), 1.78-1.83 (m, 2H), 1.56-1.61 (m, 2H), 1.22-1.44 (m, 30H). ^{13}C NMR (101 MHz, CD_3OD, δ): 167.94, 136.71, 67.73, 53.43, 49.28, 49.14, 49.00, 48.86, 48.72, 48.57, 48.43, 43.35, 42.62, 39.25, 38.85, 35.28, 31.60, 30.59, 30.55, 30.52, 30.47, 30.44, 30.39, 30.11, 30.07, 30.07, 29.25, 29.28, 29.19, 29.16, 28.91, 27.73, 27.61, 27.56, 27.21, 26.88, 25.21, 23.80, 15.98, 14.59. MS: m/z: 499.3385 ($[M-Br]^+$, calculated for $C_{27}H_{51}N_2O_2S_2^+$, 499.3386).

Kinetic. Concentrations changes over time were tracked to extract kinetic rate constants, using absorbance of DETMI-C_{16}TAB at 410 nm obtained from UV-Vis analyses. Kinetics experiments were initiated by adding NaOH to the reaction solution at time zero. Absorbance changes over time were used to calculate an observed pseudo-first order rate constant:

$$\ln(A_t - A_\infty) = \ln(A_0 - A_\infty) - kt$$

where A_t is the absorbance of solution at 410 nm at some time, t (s), A_0 is the initial absorbance of solution at 410 nm, and k (s^{-1}) is the observed rate constant obtained from the slope of the pseudo-first order rate plot. Pseudo-first order rate conditions were maintained by ensuring that the NaOH was present in excess of the initial concentration of the target analyte, DETMI-C_{16}TAB.

Experimental procedure and data processing for the determination of critical micelle concentration (CMC) using the conductivity method. Prior to the experiment, calibrate the conductivity meter using the 1413 μS/cm calibration solution supplied with the instrument; record the temperature as 26.3±0.3°C during measurement. Measure a series of prepared DETMI-C_{16}TAB aqueous solution samples of varying concentrations in ascending order of concentration. Record the data once the reading has stabilized, with three replicate measurements taken for each concentration. Principle of the automatic endpoint detection algorithm: the measurement endpoint is determined when the difference between the conductivity value of the sample under test and the average conductivity value measured by

the instrument over a 6-second period does not exceed 0.4%. Fifteen concentrations were set within the range of 1.72 μM to 860 μM to ensure complete coverage of the CMC value and to ensure sufficient concentration density for analysis near the CMC. Perform linear fits on the data sets separately, then solve the system of equations to find the point where the two lines intersect.

HRTEM sample preparation procedure. Using specialized tweezers, carefully pick up a 200-mesh carbon support film and place it face-up on a clean filter paper. Using a pipette, draw up 10 μL of DETMI-C_{16}TAB micellar solution and dispense it onto the surface of the carbon support film. Then place the carbon support film under an infrared lamp and leave it to dry completely.

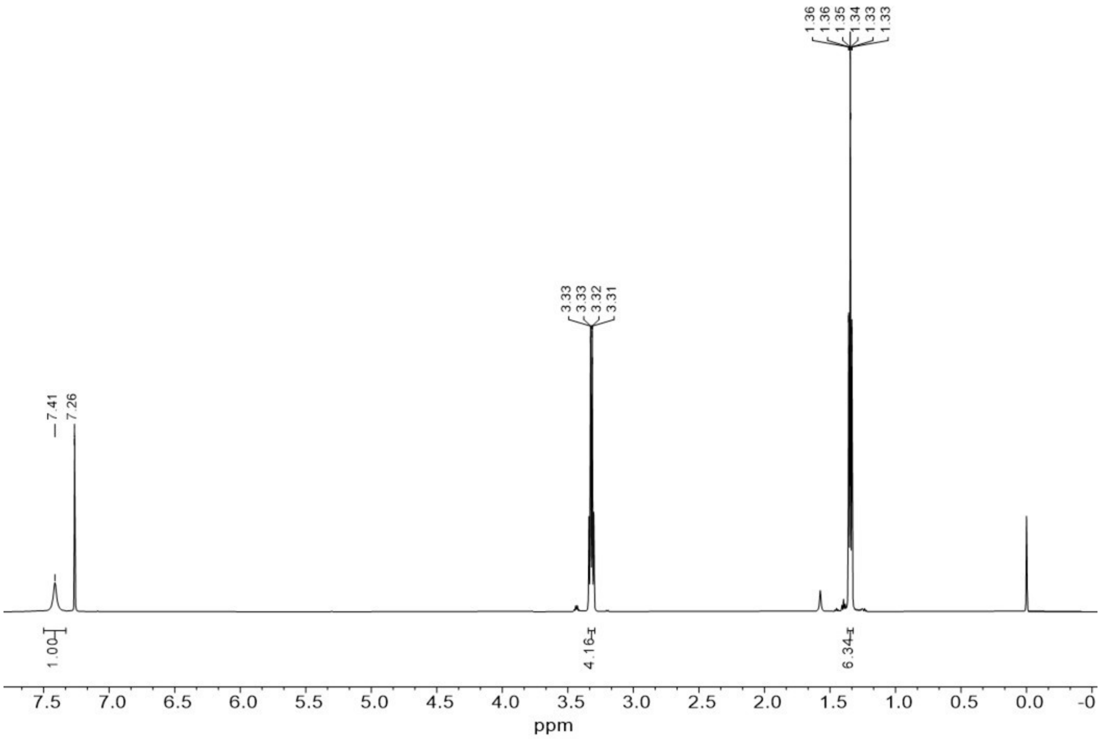

Figure S1. ^{1}H NMR spectrum of DETMI in $CDCl_3$.

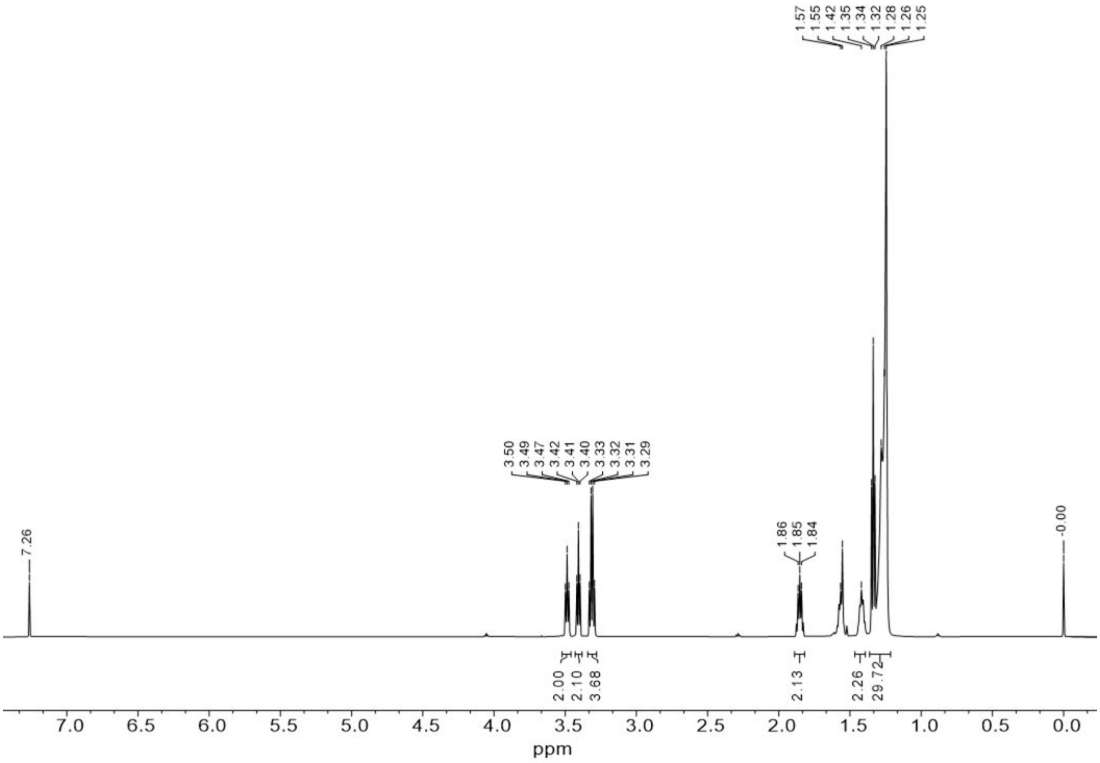

Figure S2. ^{1}H NMR spectrum of DETMI-C_{16}Br in $CDCl_3$.

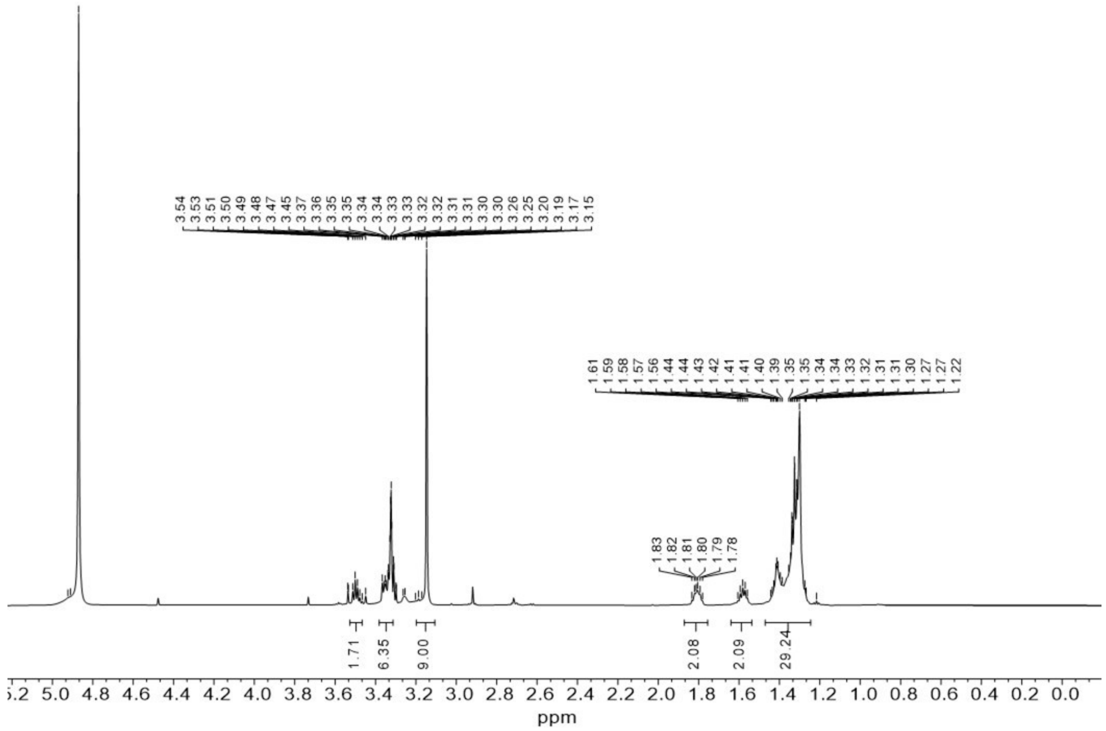

Figure S3. ^{1}H NMR spectrum of DETMI-C_{16}TAB in CD_3OD.

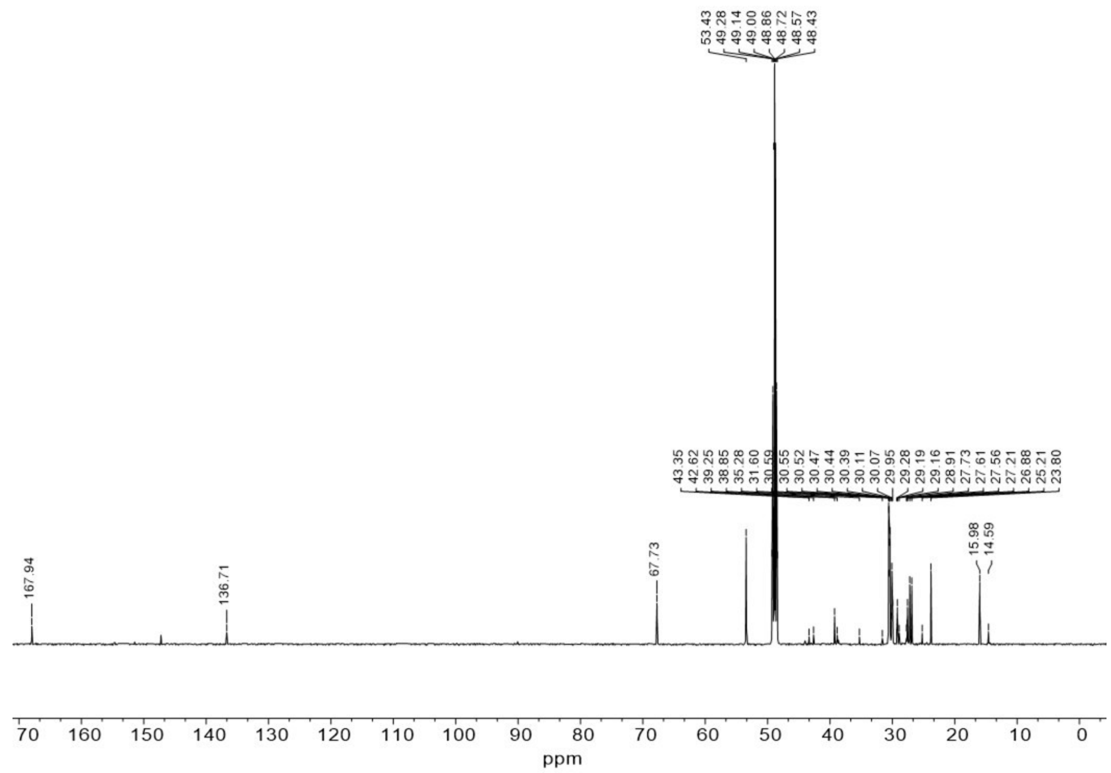

Figure S4. ^{13}C NMR spectrum of DETMI-C_{16}TAB in CD_3OD.

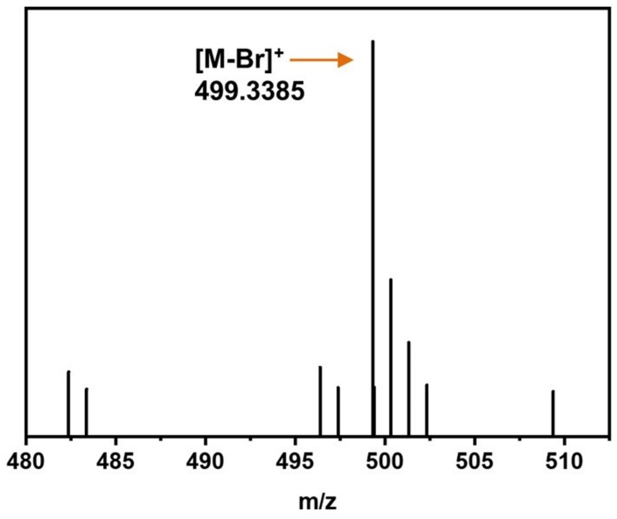

Figure S5. Magnification of the positive-ion mass spectrum of DETMI-C_{16}TAB.

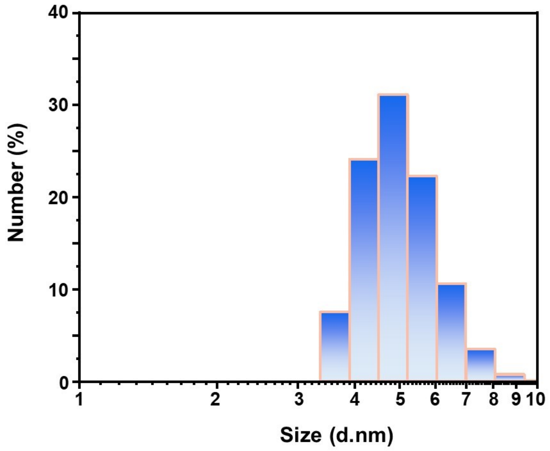

Figure S6. DLS of DETMI-C_{16}TAB micelle.

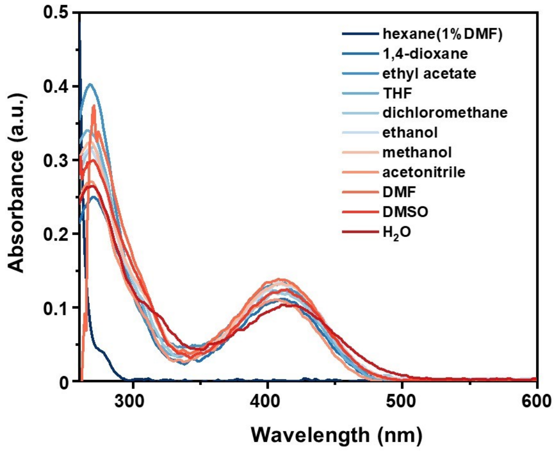

Figure S7. Absorption spectra of DETMI-C_{16}TAB with 43 μM in different solvents.

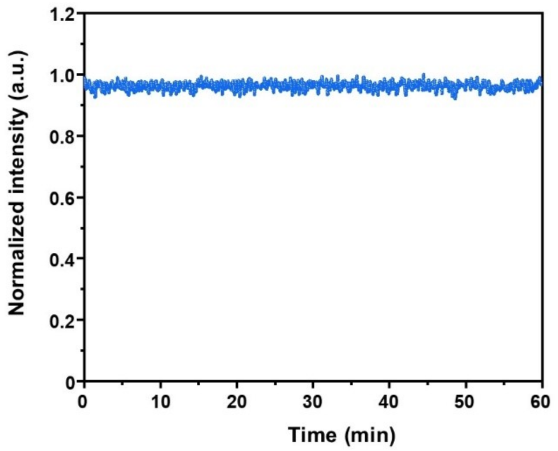

Figure S8. Photostability of the DETMI-C_{16}TAB aqueous solution (43 μM) under continuous irradiation at 410 nm using a xenon lamp.

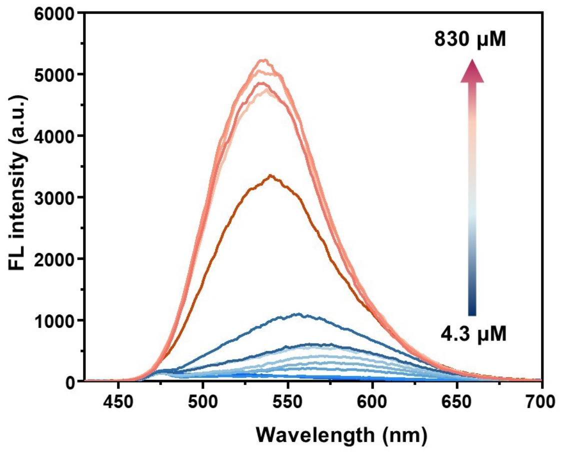

Figure S9. Fluorescence emission spectra of DETMI-C_{16}TAB with different concentrations in water.

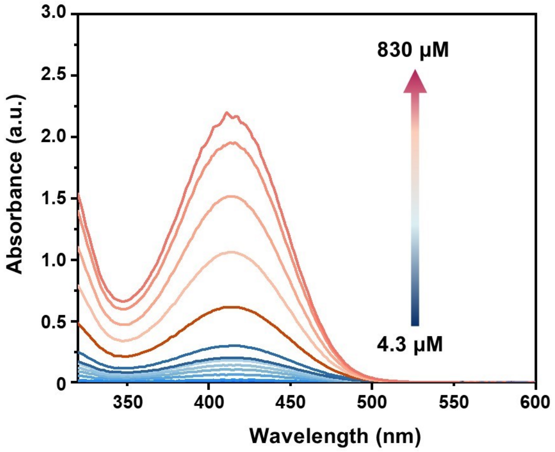

Figure S10. Absorption spectra of DETMI-C_{16}TAB with different concentrations in water.

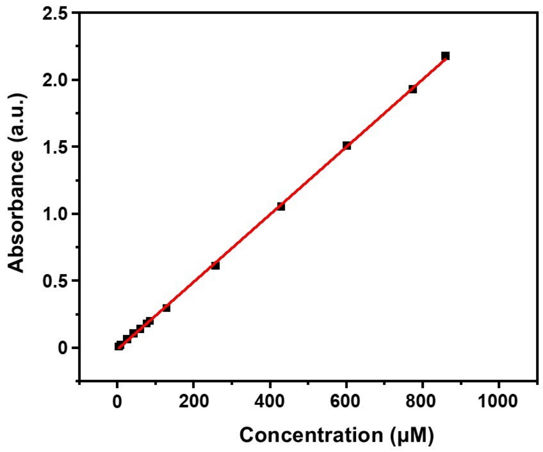

Figure S11. Plot of absorbance versus the concentrations of DETMI-C_{16}TAB.

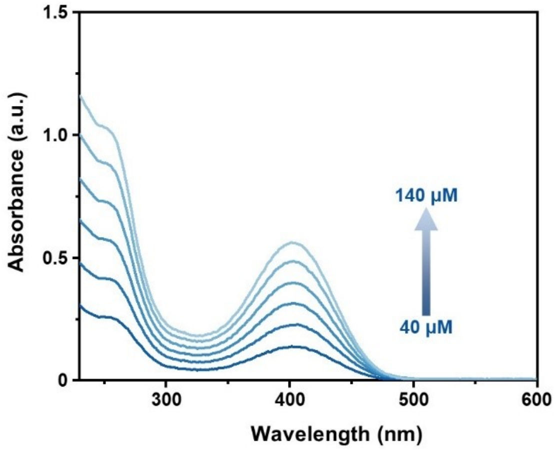

Figure S12. Absorption spectra of DETMI with different concentrations in $C_{16}TAB$ micelle.

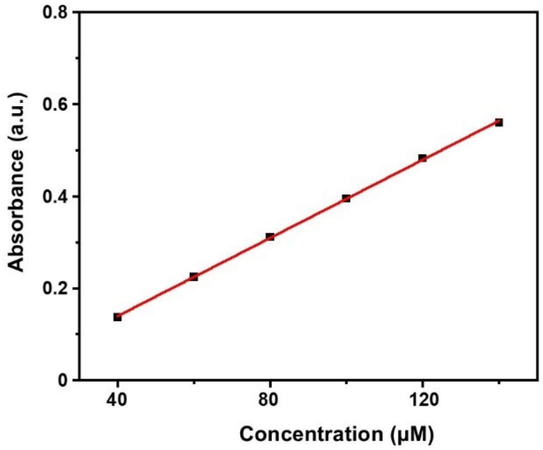

Figure S13. Plot of absorbance versus the concentrations of DETMI in $C_{16}TAB$ micelle.

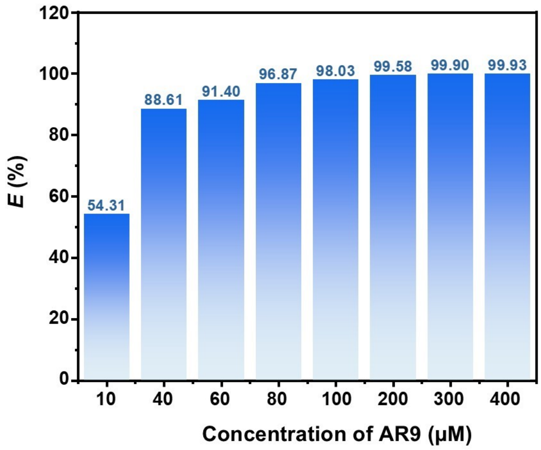

Figure S14. FRET efficiency of DETMI-C_{16}TAB micelle in the presence of AR9 with different concentrations.

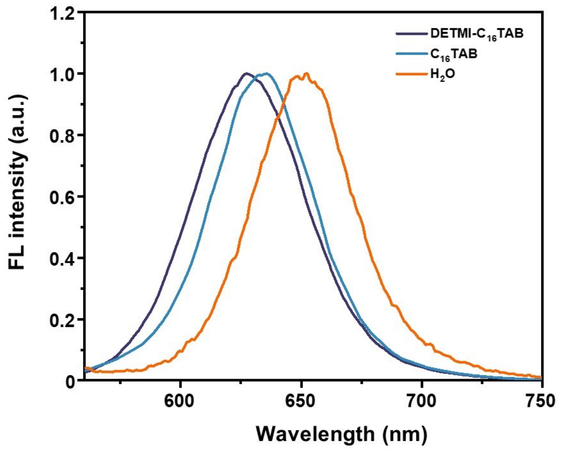

Figure S15. Normalized fluorescence emission spectra of Nile Red in DETMI-C_{16}TAB micelles, C_{16}TAB micelles and water.

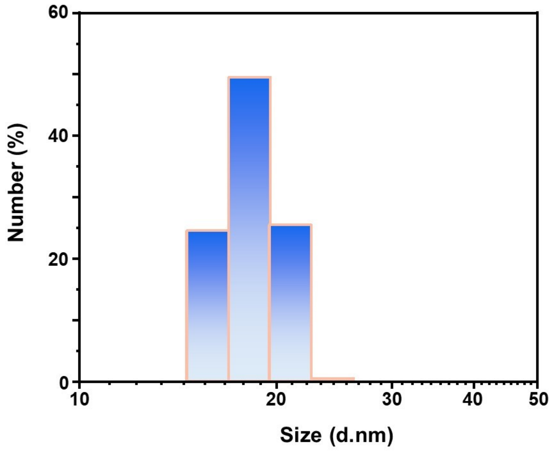

Figure S16. DLS of DETMI-C_{16}TAB micelle in 1,4-dioxane/water mixtures with 20% of 1,4-dioxane fractions.

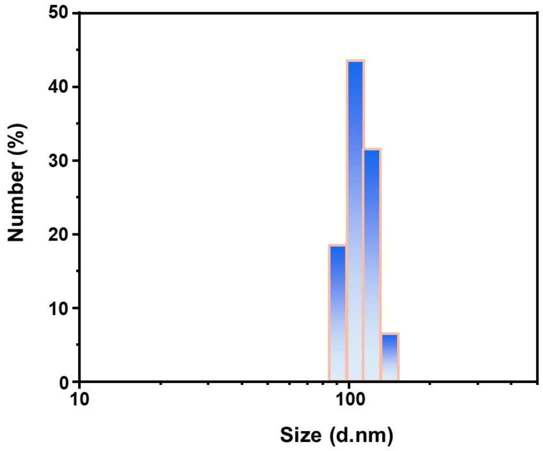

Figure S17. DLS of DETMI-C_{16}TAB micelle in acetonitrile /water mixtures with 20% of acetonitrile fraction.

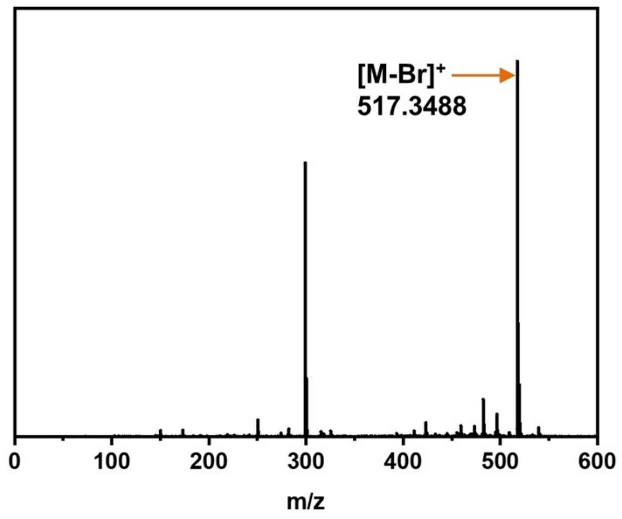

Figure S18. Positive-ion mass spectrum of the reaction product of DETMI-C_{16}TAB and OH^{-}.

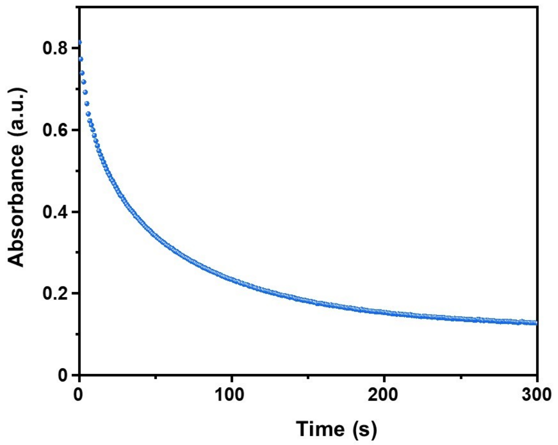

Figure S19. Kinetic traces for hydrolysis of DETMI-C_{16}TAB.

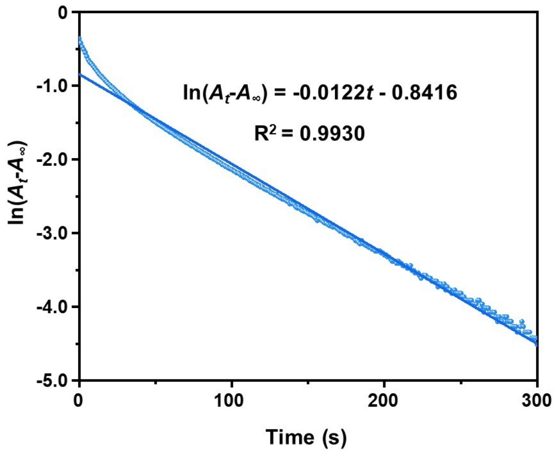

Figure S20. Plot of ln(A_t-A_∞) as a function of time for the degradation of DETMI-C_{16}TAB. The pseudo-first order rate constant k was determined from the slope of the linear regression line.

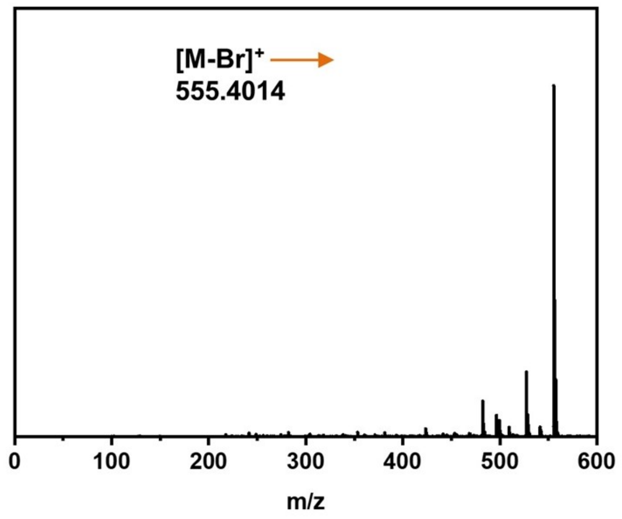

Figure S21. Positive-ion mass spectrum of the reaction product of DETMI-C_{16}TAB and C_4SH.

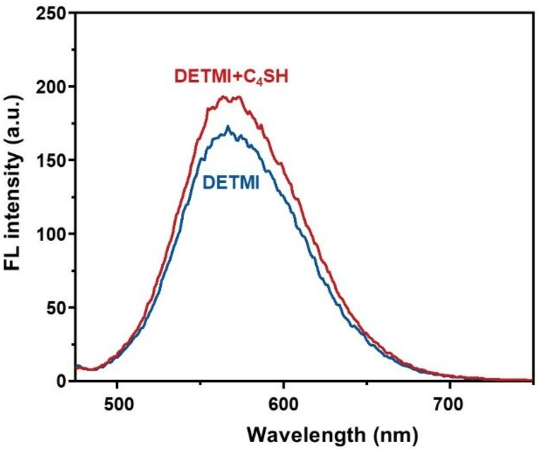

Figure S22. Fluorescence emission spectra of DETMI with addition of C_4SH.

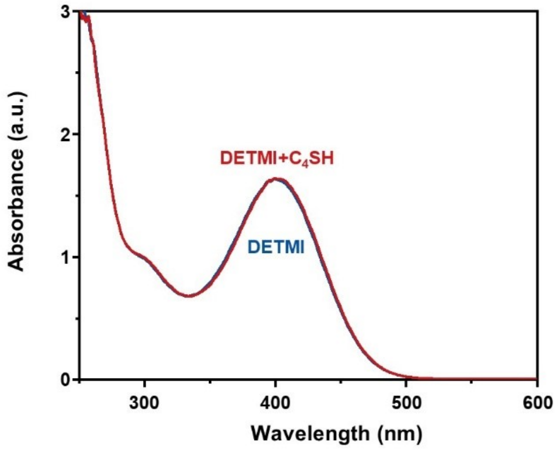

Figure S23. Absorption spectra of DETMI with addition of C_4SH.

Table S1. Maximum emission wavelength of DETMI-C_{16}TAB in different solvents.

Solvent	$E_T(30)$ (kcal·mol^{-1}) [a]	Wavelength (nm)
hexane	31	503
ethyl acetate	38.1	514
1,4-dioxane	36	517
acetonitrile	45.6	529
DMSO	45.1	535
methanol	55.4	549
H_2O	63.1	577

[a] $E_T(30)$: solvent polarity parameter;

Table S2. Solvent parameters (π^* and α) and maximum emission wavelength and of DETMI-C_{16}TAB in different solvents.

Solvent	α	π^*	Wavelength (nm)
hexane	0	-0.08	503
ethyl acetate	0	0.55	514
THF	0	0.58	514
1,4-dioxane	0	0.55	517
dichloromethane	0.3	0.82	529
acetonitrile	0.19	0.75	529
DMF	0	0.88	528
DMSO	0	1	535
ethanol	0.83	0.54	545
methanol	0.93	0.6	549
H_2O	1.17	1.09	577

Solvent parameters (π^* and α) are taken from (J. Org. Chem. 1983, 48, 2877).

Table S3. Multiple linear regression results for the Kamlet–Taft solvatochromic analysis of DETMI-C_{16}TAB.

Parameter	Coefficient (cm^{-1})	Std. Error	*t*-Value	*p*-Value
Intercept	19923.0	92.9	214.5	< 0.001
α	−1193.2	93.9	−12.7	< 0.001
π^*	−1044.8	133.7	−7.8	< 0.001

$n = 11$, $R^2 = 0.9682$, SE = 126.5 cm^{-1}.

Table S4. The pseudo-first-order rate constant (k) for $C_{16}TAB$ micelle-catalyzed hydrolysis reactions in the relevant literature.

k	Reaction	Reference
$2–150\times10^{-2}$ s^{-1}	hydrolysis of 1,3-benzoxazine-2,4-dione and its derivatives	J. Colloid Interface Sci. 361 (2011) 205–211
1.23×10^{-3} min^{-1}	carbaryl hydrolysis	Pharm. Res. 8, 1155–1158 (1991).
4.4×10^{-3} s^{-1}	spontaneous hydrolysis of phenyl chloroformate	Langmuir 2003, 19, 7206-7213
40.7×10^{-3}–101.5×10^{-3} s^{-1}	alkaline hydrolysis of malachite green	J. Phys. Chem. B 2020, 124, 2048−2059
